# Limitations of Antibiotic MIC-Based PK-PD Metrics: Looking Back to Move Forward

**DOI:** 10.3389/fphar.2021.770518

**Published:** 2021-10-29

**Authors:** Cornelia B. Landersdorfer, Roger L. Nation

**Affiliations:** Drug Delivery, Disposition and Dynamics, Monash Institute of Pharmaceutical Sciences, Monash University, Parkville, VIC, Australia

**Keywords:** antibiotics, PK-PD relationships, MIC-based metrics, limitations and implications, model-informed precision dosing

## Abstract

Within a few years after the first successful clinical use of penicillin, investigations were conducted in animal infection models to explore a range of factors that were considered likely to influence the antibacterial response to the drug. Those studies identified that the response was influenced by not only the total daily dose but also the interval between individual doses across the day, and whether penicillin was administered in an intermittent or continuous manner. Later, as more antibiotics were discovered and developed, antimicrobial pharmacologists began to measure antibiotic concentrations in biological fluids. This enabled the linking of antibacterial response at a single time point in an animal or *in vitro* infection model with one of three summary pharmacokinetic (PK) measures of *in vivo* exposure to the antibiotic. The summary PK exposure measures were normalised to the minimum inhibitory concentration (MIC), an *in vitro* measure of the pharmacodynamic (PD) potency of the drug. The three PK-PD indices (ratio of maximum concentration to MIC, ratio of area under the concentration-time curve to MIC, time concentration is above MIC) have been used extensively since the 1980s. While these MIC-based summary PK-PD metrics have undoubtedly facilitated the development of new antibiotics and the clinical application of both new and old antibiotics, it is increasingly recognised that they have a number of substantial limitations. In this article we use a historical perspective to review the origins of the three traditional PK-PD indices before exploring in detail their limitations and the implications arising from those limitations. Finally, in the interests of improving antibiotic development and dosing in patients, we consider a model-based approach of linking the full time-course of antibiotic concentrations with that of the antibacterial response. Such an approach enables incorporation of other factors that can influence treatment outcome in patients and has the potential to drive model-informed precision dosing of antibiotics into the future.

## Introduction

Around the middle of the 20th century there was a growing realisation of the need to extend beyond the basic concept of the dose-response relationship for a medicine administered to a patient, to recognise that the driving force of the biological response was further downstream. This initiated the move towards linking response with a drug concentration in a biological fluid and, as reviewed by others, this was the beginning of the pharmacokinetic-pharmacodynamic (PK-PD) era (Hochhaus et al., 2000; Csajka and Verotta, 2006). Pleasingly, antimicrobial pharmacologists were early and active participants in this field. Their research resulted in important insights into the nature of the PK-PD relationship describing the link between the *in vivo* exposure to an antibiotic and the resultant antibacterial effect. Understanding that relationship is critical in the nonclinical and clinical development of a new antibiotic because it guides the dosage regimens to be evaluated in clinical trials and subsequently the regimens included in the product information at the time of regulatory approval of the antibiotic product (European Medicines Agency, 2016; Food and Drug Administration, 2017). In the clinical use of the antibiotic after its approval, knowledge of the PK-PD relationship enables the monitoring of antibiotic exposure in a patient being treated, most commonly assessed by measurement of the drug concentration in plasma, and titration of the dosage regimen to achieve a level of exposure that is considered likely to maximise bacterial killing and minimise the emergence of resistance to the antibiotic (Huttner et al., 2015; Wicha et al., 2021).

In this review we briefly look back at key events in the birth and development of the discipline of antibiotic PK-PD. In particular, we focus on a key product of that development, three so-called PK-PD indices, that have been the most common way to link antibiotic PK exposure and antibacterial PD response in studies conducted since the late 1980s. Next, we consider in detail major limitations of those PK-PD indices, and a number of important implications associated with the limitations. Finally, in the interests of moving forward and fostering the ongoing evolution of the discipline, we consider alternative approaches that offer very substantial promise of enhancing the scientific approach to the development and rational clinical use of antibiotics.

### Antibiotic PK-PD: Looking Back

The first reports of the successful parenteral and oral administration of penicillin to infected patients occurred during World War II (Fleming, 1943; Florey and Florey, 1943). Only a few years later, visionary scientists published the results of studies conducted in animal infection models in which they investigated a range of factors that they considered may influence the antibacterial response to penicillin, the first clinically used β-lactam antibiotic (Jawetz, 1946; Eagle et al., 1947; Schmidt et al., 1949; Eagle et al., 1950a; Eagle et al., 1950b; Eagle et al., 1952; Eagle et al., 1953). Harry Eagle was especially active in this regard. He conducted elegant studies that showed that the survival of animals with a range of different experimental infections was dependent on not only the dose of penicillin but also the dosing interval, as well as the site and duration of infection, the immune status of the host and also the bacterial inoculum (Eagle et al., 1947; Eagle et al., 1950b; Eagle et al., 1953). Although Eagle did not measure the penicillin concentration in the animals, he clearly understood the temporal pattern of *in vivo* exposure to the antibiotic arising from different dosing regimens. Based on his experimental observations, he stated that “the primary determinant of therapeutic activity is the time for which the drug remains at effective concentrations at the focus of infection” and, remarkably, suggested that continuous infusion of penicillin was likely to be the most effective way to administer the drug to patients (Eagle et al., 1953). In much more recent times, that mode of administration for other β-lactam antibiotics has been reported to be associated with decreased hospital mortality compared with intermittent dosing in critically ill patients with severe sepsis (Roberts et al., 2016).

The studies conducted by Eagle and the other antimicrobial pharmacology pioneers were the genesis of the concept of PK-PD relationships of antibiotics that led to the defining of time- and concentration-dependent antibacterial activity (Eagle et al., 1953; Vogelman and Craig, 1986). Subsequently, William Craig’s group reported the first dose-ranging and dose-fractionation PK-PD studies in a neutropenic mouse-thigh infection model (Vogelman et al., 1988). Using that experimental approach, the investigators sought to differentiate among three potential PK-PD indices of antibacterial activity. The indices are the ratio of the maximum plasma antibiotic concentration to the minimum inhibitory concentration (i.e., C_max_/MIC), the ratio of the area under the plasma concentration-time curve to the MIC (i.e., AUC/MIC), and the percentage of time for which the plasma concentration exceeds the MIC (i.e., %T_>MIC_). Those extensive landmark studies identified that the antibacterial activity of aminoglycosides was best predicted by AUC/MIC, while for β-lactams the antibacterial activity was correlated with %T_>MIC_ (Vogelman et al., 1988). Since then, many similar studies have identified the relevant PK-PD index of numerous antibiotics across a range of classes, and that information has been used extensively in their development and clinical use (Craig, 1998; Ambrose et al., 2007; Roberts et al., 2014; Rawson et al., 2021).

In submissions for approval of new antibiotics, regulatory agencies require inclusion of data from nonclinical PK-PD studies (European Medicines Agency, 2016; Food and Drug Administration, 2017). The most predictive nonclinically derived PK-PD index and the target value of that index for various magnitudes of bacterial kill (usually stasis, or 1- or 2-log_10_ reduction in counts of viable bacterial cells) have been used for many years. The uses include: informing of many important steps in the development of new antibiotics, especially the choice of dosage regimens to be examined in clinical studies (Friberg, 2021); determining susceptibility breakpoints (Turnidge and Paterson, 2007; Mouton et al., 2012); and, translating to the clinical setting to select a plasma exposure target for the routine care of patients (Roberts et al., 2014; Wicha et al., 2021). The use of the PK-PD indices in these ways has undoubtedly been very helpful across the range of areas where they have been applied, as they have provided a way to semi-quantitatively describe the exposure-response relationship of antibiotics. However, it is increasingly recognised that there are a number of important limitations associated with the use of these traditional PK-PD indices (Seeger et al., 2021a; Friberg, 2021; Landersdorfer and Nation, 2021). Some of the limitations relate to the nature of the nonclinical experimental models and approaches that are used to establish what is concluded to be the most predictive index, while other limitations arise from the use, in each of the indices, of the MIC as a measure of the antibacterial “potency” of the antibiotic. Each of these clusters of limitations is discussed below, as are some important implications associated with the limitations.

### Limitations of Traditional Antibiotic PK-PD Indices

#### Problems With the Way in Which PK-PD Indices are Determined

Fundamentally, each of the PK-PD indices is an attempt to link a summary PK measure of exposure (e.g., C_max_, AUC) with a measure of the PD “potency” of the antibiotic as provided by the MIC determined *in vitro*. An appreciation of the limitations of these PK-PD indices requires an understanding of the way in which the indices and the associated target values for various magnitudes of bacterial kill are determined.

**FIGURE 1:**
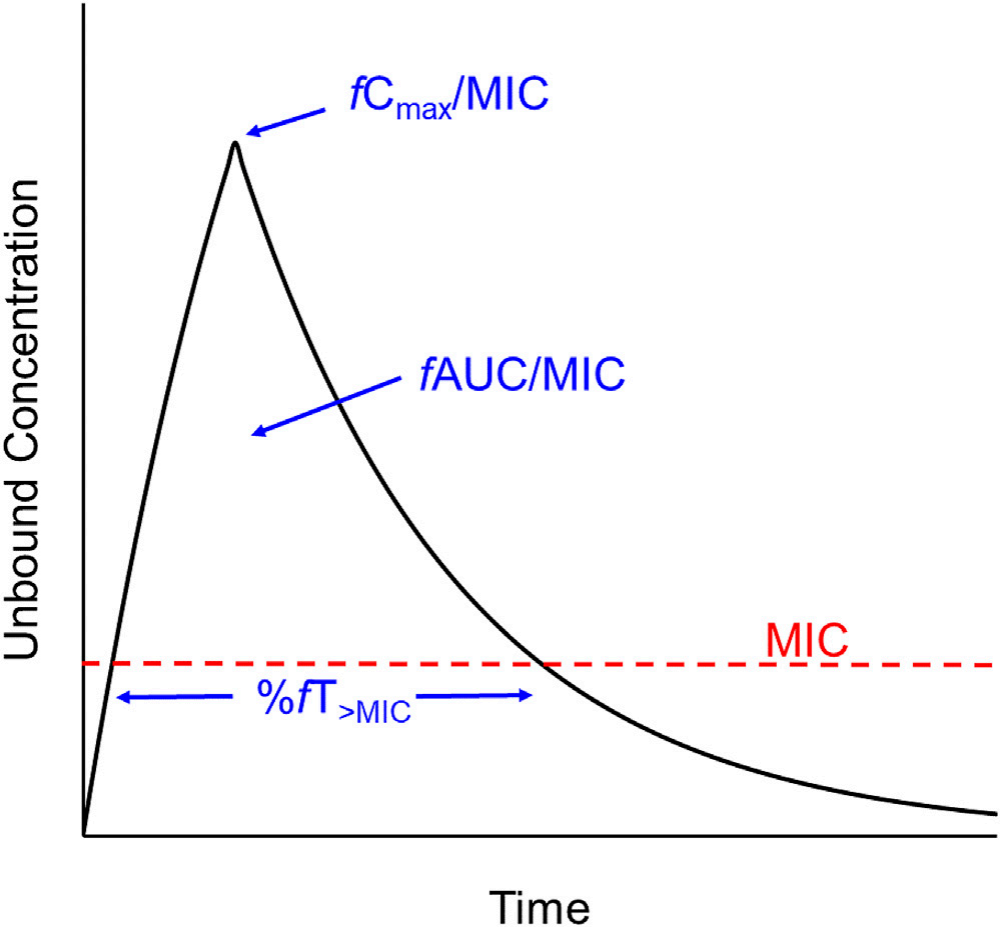
Hypothetical concentration vs. time profile of an antibiotic showing the three traditional, MIC-based PK-PD indices described in the text.

**FIGURE 2:**
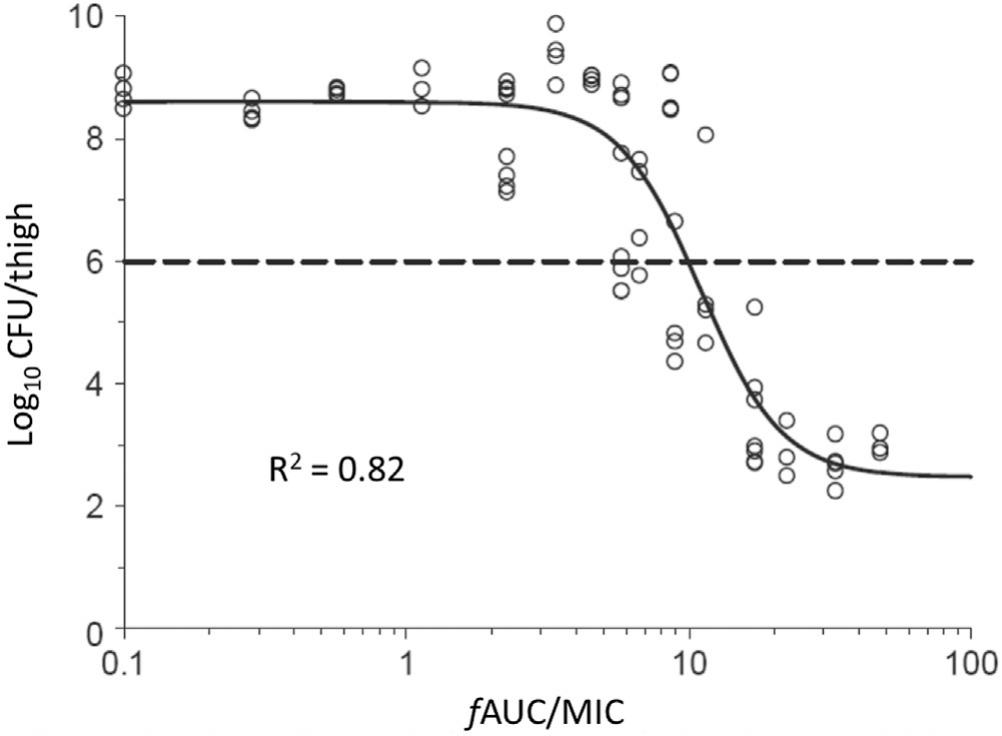
Relationship for *Pseudomonas aeruginosa* ATCC 27853 between the log_10_ colony forming units (CFU) per thigh at 24 h and *f*AUC/MIC of colistin (Cheah et al., 2015). Each symbol represents the value from a single thigh. The data points on the *y*-axis are for untreated (control) mice at 24 h after commencement of therapy in the animals treated with colistin. The dotted line represents the average bacterial burden in the thighs at the start of colistin treatment. The figure is panel “a” of Figure 3 in the original publication of Cheah et al., 2015.

Nonclinical PK-PD studies are usually conducted in small animal infection models or *in vitro* dynamic infection models (the latter are referred to as “dynamic” as they allow simulation of concentration-time profiles as occur in patients with various PK properties and after different modes of administration) (Gloede et al., 2010; Velkov et al., 2013). The neutropenic mouse thigh and lung infection models have been most commonly used. In these models, mice are rendered neutropenic by administration of cyclophosphamide. The neutropenic state facilitates establishment of a robust infection and also enables examination of the activity of the antibiotic in the relative absence of immune function effects. Subsequently, mice are infected in the thigh or lungs by introducing a known inoculum [10^5^–10^8^ colony forming units (CFU)] of the microorganism under investigation; at least three, and preferably more, bacterial strains should be studied for each antibiotic-species pair (Zhao et al., 2016; Jorda and Zeitlinger, 2020). After 1–2 h, antibiotic treatment commences with a number of different regimens used in groups of animals. Dose-ranging and dose-fractionation regimens are used such that the total daily dose spans a wide range and, at a given daily dose, various fractions of the total daily dose are administered at different intervals (e.g., 10 mg/kg once daily, 5 mg/kg 12 hourly, 2.5 mg/kg 6 hourly, 1.25 mg/kg 3 hourly). The dose fractionation is intended to enable differentiation across the three PK-PD indices, by untangling the interdependence of C_max_/MIC, AUC/MIC and %T_>MIC_ if the same dosing interval was used for each total daily dose (Craig, 1998). After 24 h of treatment, mice are euthanised and viable bacteria are enumerated and expressed as CFU/thigh or CFU/lung. From PK studies over a relevant range of doses in neutropenic infected mice, the C_max_/MIC, AUC/MIC and %T_>MIC_ for each of the numerous dosage regimens above can be determined. Concentration-ranging studies on the protein binding of the antibiotic in the plasma of neutropenic infected mice enable determination of the unbound (free) fraction of the drug. This information is used to convert each PK-PD measure to the corresponding index referenced to the microbiologically active free drug i.e. *f*C_max_/MIC, *f*AUC/MIC and %*f*T_>MIC_ as shown in Figure 1. The viable counts (CFU/thigh or CFU/lung at 24 h) for each of the dosage regimens and the corresponding value of each index are then subjected to analysis *via* the sigmoidal maximum effect (E_max_) function, and the index with the highest statistical correlation to the antibacterial response is considered to be the most predictive PK-PD index. As an example, Figure 2 presents the relationship for *Pseudomonas aeruginosa* ATCC 27853 between the log_10_ CFU/thigh at 24 h and *f*AUC/MIC of colistin; of the three PK-PD indices, *f*AUC/MIC was the most predictive of antibacterial activity as assessed by CFU counts at 24 h (Cheah et al., 2015). The most predictive index arising from such analyses can then be used along with information on MIC distributions for clinical isolates of the relevant bacterial species (European Committee on Antimicrobial Susceptibility Testing, 2021), relative magnitudes of plasma unbound fraction in mice and humans, and information on population PK and concentration-related toxicities in patients to: 1) determine a susceptibility breakpoint for the bacterial species; 2) propose a target *in vivo* exposure in humans; and, 3) estimate probabilities of target attainment for various clinical dosage regimens of the antibiotic (Ambrose et al., 2007; Mouton et al., 2012).

There are several important limitations related to the traditional PK-PD indices, a number of which occur because only one assessment time point, usually at 24 h, is used to quantify the impact of treatment on the total bacterial population (Friberg, 2021; Landersdorfer and Nation, 2021). The first limitation arises because the time-course of bacterial response to a treatment is dictated by the balance of biological processes including the natural growth and death of bacteria, the killing of bacteria mediated by the antibiotic and any regrowth of bacteria. A single time point approach to assessing antibacterial effect provides no information on the time-course of the response and whether bacterial numbers are increasing or decreasing at the time at which antibacterial response is determined. Secondly, assessing response at 24 h provides little opportunity to gauge the possible emergence of resistance, as that may only become evident over a longer time. Thirdly, and related to the preceding point, bacterial response is almost always based on viable counting of the total bacterial population, without attempts to quantify less-susceptible bacterial subpopulations; yet it may be amplification of less-susceptible subpopulations that negatively impact the response at later times (Andersson et al., 2019).

Not surprising in view of the above considerations, it has become increasingly evident that the choice of the most predictive traditional PK-PD index and the magnitude of that index required for a certain level of bacterial kill or resistance suppression may be influenced by a number of factors. These include the characteristics of an isolate, for example whether the isolate is hypermutable [i.e., has vastly increased mutation rate (Oliver et al., 2000)] or heteroresistant [i.e., contains covert resistant bacterial subpopulations within an isolate that is categorised as “susceptible” based upon MIC testing (Cheah et al., 2015; Andersson et al., 2019; Dewachter et al., 2019)]. Other factors that can influence the predictive ability of the three PK-PD indices are the PK in the patient group of interest and the mode of administration which can affect the shape of the PK exposure profile (Rees et al., 2015; Kristoffersson et al., 2016; Rees et al., 2016; Landersdorfer et al., 2018).

A simulation-based evaluation replicated *in silico* a murine dose-fractionation study of meropenem to examine the sensitivity of the “most predictive” PK-PD index to experimental design (e.g., bacterial inoculum, antibiotic dosing regimen), drug susceptibility and different PK profiles (Kristoffersson et al., 2016). It is important to note that the PK characteristics of antibiotics in mice differ substantially from those in humans (e.g., mice usually have higher clearance per unit of body weight and shorter elimination half-life). Reassuringly, the data simulated using a semi-mechanistic model, also known as a mechanism-based model, agreed well with the published murine model observations. With both the original analysis of the murine model data (Sugihara et al., 2010) and the *in silico* simulations of that study (Kristoffersson et al., 2016), antibiotic efficacy was much more highly correlated with %*f*T_>MIC_ than with either *f*C_max_/MIC or *f*AUC/MIC, and similar magnitudes of %*f*T_>MIC_ were required for 2-log kill of bacteria in the murine and *in silico* analyses. However, the simulations found that when dosing frequency in mice was increased, *f*AUC/MIC became an equal or superior predictor of efficacy compared to %*f*T_>MIC_. When human PK properties were used as input to the semi-mechanistic model, %*f*T_>MIC_ and *f*AUC/MIC had similar predictive capacities, with preference for the former index when the half-life was short and the latter index when half-life was long. These findings indicate that the “most predictive” PK-PD index is not necessarily the same across all possible scenarios. In addition, the magnitude of the index associated with a given extent of bacterial kill was sensitive to the conditions. For example, in simulated patients with augmented renal clearance 47%*f*T_>MIC_ was required for 2-log_10_ bacterial kill at 24 h based on the murine infection model data, while for patients with impaired renal function the corresponding value was 73%*f*T_>MIC_ (Kristoffersson et al., 2016). Interestingly, the PK-PD target from the actual experimental murine studies (45%*f*T_>MIC_) (Sugihara et al., 2010) and both of the simulated human values above are now generally regarded as too low for treatment of infections in critically ill patients where a PK-PD target of at least 100%*f*T_>MIC_ is commonly used (Huttner et al., 2015). This apparent disparity in the magnitude of the PK-PD index may be the result of failure in the murine model studies to consider the magnitude and shape of the exposure profile required to suppress resistance.

**FIGURE 3:**
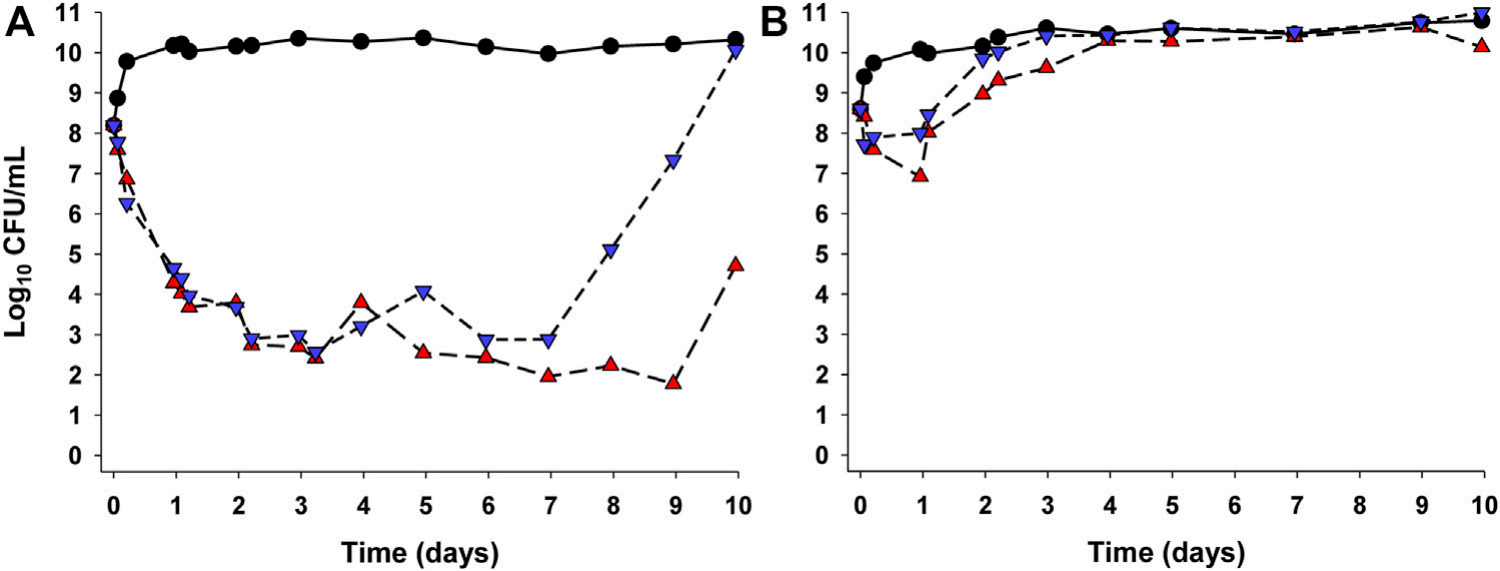
Bacterial counts (CFU/mL) of *Pseudomonas aeruginosa* wild-type reference strain PAO1 which is non-hypermutable **(A)** and its PAOΔ*mutS* isogenic hypermutable strain **(B)** in the hollow-fibre *in vitro* infection model over 10 days (Landersdorfer et al., 2018). Each strain had a meropenem MIC of 1 mg/L and each was exposed to two meropenem dosing regimens (3 g/day as a continuous infusion [red triangles] generating exposure of 100%*f*T_>5×MIC_ and 1 g as 30 min intravenous infusion every 8 h [inverted blue triangles] generating exposure of 36%*f*T_>5×MIC_). The respective growth controls are shown as black circles. The figure is adapted from Figure 4 in the original publication of Landersdorfer et al., 2018.

Whether or not a bacterial strain is hypermutable can also affect the applicability of traditional PK-PD indices, as demonstrated by studies in a hollow-fibre *in vitro* infection model (HFIM) (Landersdorfer et al., 2018). That study involved *P. aeruginosa* wild-type reference strain PAO1 which is non-hypermutable and its isogenic hypermutable strain PAOΔ*mutS*, each with a meropenem MIC of 1 mg/L. When those strains were each subjected in the HFIM to two different meropenem dosing regimens (1 g every 8 h as 0.5 h infusions and 3 g daily as continuous infusion) over 10 days, the response to each regimen was vastly inferior for the hypermutable strain. This was the case even though the MIC values were the same for both strains, and the intermittent infusion and continuous infusion regimens achieved identical exposures (36%*f*T_>5×MIC_ and 100%*f*T_>5×MIC_, respectively) against each strain (Figure 3). In addition, quantification of resistant subpopulations revealed that continuous infusion of meropenem was able to suppress resistance emergence for the non-hypermutable wild-type strain but not for the hypermutable strain (Landersdorfer et al., 2018). Heteroresistance is another bacterial characteristic that can confound PK-PD relationships. A study investigating the PK-PD relationship of colistin against *Acinetobacter baumannii* in a murine lung infection model involved three strains, all of which were categorised as “susceptible” at baseline based on MIC testing (Cheah et al., 2015). However, population analysis profiles (i.e., studies investigating the ability of colonies to grow on agar containing various concentrations of colistin) revealed that one of the strains with MIC 1 mg/L was devoid of resistant subpopulations, while the remaining two strains were heteroresistant (MICs of 0.5 and 1 mg/L). In the murine lung infection model, the susceptible, non-heteroresistant strain responded to colistin treatment at tolerated daily doses as evidenced by approximately 2-log_10_ of bacterial kill; however, with the two heteroresistant strains there was no decrease in bacterial numbers in lungs across a wide range of *f*AUC/MIC values, even at the highest tolerated dosage regimen of colistin (Cheah et al., 2015). In other work it has been shown that at a given *f*AUC/MIC of tobramycin or ciprofloxacin against *P. aeruginosa*, the rate and extent of bacterial kill and emergence of resistance are influenced by the shape of the exposure profile (Rees et al., 2015; Rees et al., 2016). The results of these four studies are not what would be expected if the respective MIC-based PK-PD index (%*f*T_>MIC_ or *f*AUC/MIC) provided a generally applicable link between PK exposure and PD response.

Even before considering the substantial limitations associated with the use of MIC within each index, it is clear that the three traditional PK-PD metrics can be confounded by a number of factors, and may not always be able to predict the optimal magnitude and shape of the exposure profile to achieve a desired antibacterial outcome in a patient i.e. maximal bacterial kill and no or minimal emergence of resistance. In large measure these deficiencies in the PK-PD indices are the result of: 1) PD response in the nonclinical infection model being assessed at a single, relatively early, time point; 2) use of summary PK metrics (e.g., C_max_, AUC) that ignore the shape of the exposure profile; and, 3) failure to recognise and capture information on bacterial characteristics such as hypermutability and heteroresistance. Importantly, the three traditional PK-PD indices do not incorporate factors other than antibacterial effect that may have an important influence on the clinical outcome in a treated patient. Such factors include the immune status, presence of comorbid conditions and severity of overall illness of the patient. Clearly, the limitations discussed above arise even when antibiotics are used in monotherapy regimens, and an additional substantial limitation is that the three indices cannot be used to optimise the exposure profile of each antibiotic in combination therapy (Couet, 2018).

### MIC: A Highly Problematic Measure of Antibacterial PD Activity

MIC is included as the “PD” component in each of the three traditional PK-PD indices, but it is important to recognise that it has a number of limitations that go well beyond the fact that it takes a day or more for its estimation (Wen et al., 2016; Mouton et al., 2018a; Bader et al., 2018; Mouton et al., 2018b; Mouton, 2018; Seeger et al., 2021a; Kowalska-Krochmal and Dudek-Wicher, 2021). The MIC is a very crude *in vitro* measure of the “potency” of an antibiotic that is determined in a matrix bearing little resemblance to the physiological milieu at an infection site in a patient. A low inoculum is used in the measurement of MIC; for example, in the broth microdilution assay, a small volume of an inoculum of 5 × 10^5^ CFU/mL is used which may be quite different from the bacterial density prevailing in an infected patient (Migiyama et al., 2021). In addition, the small total number of bacterial cells placed into each microplate well lowers the probability of less-susceptible subpopulations being present at the start of the incubation. However, if resistant cells are indeed present in an isolate comprised mostly of susceptible cells, the combination of the relatively short incubation time (16–20 h) and the blunt endpoint (visual perception of turbidity only being evident above ∼10^7^–10^8^ CFU/mL) converge to conceal covert resistance, i.e., heteroresistance (Andersson et al., 2019; Dewachter et al., 2019). Even in the absence of resistant subpopulations, lack of turbidity does not mean there are no viable bacteria present at the end of the incubation period. Importantly, as with the animal infection models discussed above, an MIC measurement is based on an evaluation at a single time point, therefore the MIC does not reveal the rate and extent of bacterial kill nor any regrowth that may have occurred. The above-mentioned limitations were nicely summarised by Eagle in 1953 in his ground-breaking work on penicillin when he stated that “such ‘sensitivity’ tests determine not the concentration of penicillin that kills the particular organism at the fastest possible rate, but rather the concentration that prevents visible growth under the arbitrary conditions of the particular test” (Eagle et al., 1953).

In addition to the considerations above, MIC assays are conducted using a series of 2-fold dilutions of antibiotic, so even in a perfect world without errors MIC values would be reported in 2-fold steps. In reality, MIC tests are subject to very poor accuracy and precision within and across laboratories; results of replicate assays for a given isolate may range across two or more 2-fold dilutions (Mouton et al., 2018a; Mouton et al., 2018b). Imprecision and poor accuracy do not present a major problem for the strains used in animal infection models and other nonclinical studies because the MIC values used in calculating the PK-PD indices discussed above are based upon replicate determinations. However, it is still important to recognise that the values of MIC-based PK-PD metrics for a particular antibiotic and strain pair from such a nonclinical infection model are not continuous in nature (i.e., they are stepped values) because of the 2-fold dilutions of antibiotic concentrations used in estimation of the MIC within each index. This problem is minimized in animal infection model or dynamic *in vitro* infection model studies aimed at elucidating the predictive PK-PD index and target values for various magnitudes of bacterial kill by ensuring that several bacterial strains are included in the study to inform setting of PK-PD targets and breakpoints (Zhao et al., 2016; Mouton et al., 2018b; Jorda and Zeitlinger, 2020). However, an inaccurate estimate from a single determination of MIC for an isolate from a particular patient is problematic in the clinical application of MIC-based PK-PD targets within the framework of what has been described as therapeutic drug monitoring (TDM) (Mouton et al., 2018b; Roberts et al., 2019; Märtson et al., 2020; Seeger et al., 2021a; Goutelle et al., 2021; Jorgensen et al., 2021; Moser et al., 2021). Thus, if an isolate from an infected patient returns an MIC estimate from an assay which has a typical error range of two dilutions (i.e., potentially a 4-fold range of reported MIC values), it follows that the PK-PD driven target plasma concentration for that patient would vary 4-fold if the PK-PD index for the antibiotic is *f*AUC/MIC or *f*C_max_/MIC. Clearly, if the “true” MIC of the isolate is at the top of the error range but the reported MIC is at the bottom of the range, there is substantial risk of under dosing the patient. If the converse applied for an antibiotic of low therapeutic index, there may be increased risk of treatment-related toxicity if the reported MIC resulted in the decision to increase the dosage regimen (Märtson et al., 2020; Goutelle et al., 2021). Either of these outcomes may decrease the probability of achieving a good clinical outcome for the patient.

### Antibiotic PK-PD: Moving Forward

Although the traditional PK-PD indices have undoubtedly been helpful in facilitating preclinical development of antibiotics, their translation into clinical studies and ultimately in proposing dosage regimens for various categories of patients, the indices are associated with several substantial limitations as discussed above. The need to capture the full time-course of both the PK of the antibiotic and the PD of its antibacterial effect, without reliance on MIC, has been increasingly recognised; fortunately, moves in this direction have already commenced (Seeger et al., 2021a; Friberg, 2021; Landersdorfer and Nation, 2021; Wicha et al., 2021).

**FIGURE 4:**
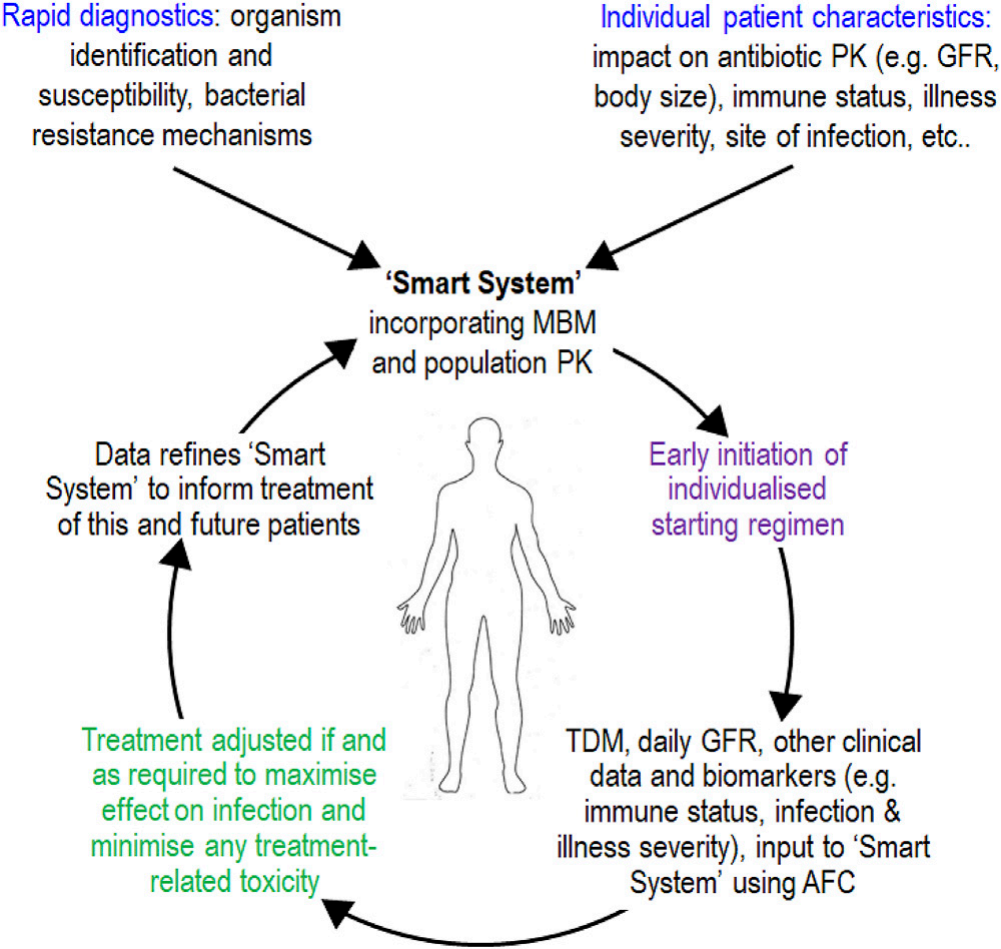
Schematic of model-informed precision dosing (MIPD) achieved *via* a so-called “Smart System” (Landersdorfer and Nation, 2021). Such a system may inform delivery of future precision antibiotic therapy and improve treatment of infections, especially in critically ill patients with sepsis or septic shock who have unstable pathophysiology and difficult-to-treat infections. AFC, adaptive feedback control; GFR, glomerular filtration rate; MBM, mechanism-based model; PK, pharmacokinetic; TDM, therapeutic drug monitoring (antibiotic concentration in plasma or, if possible, at the site of infection). The figure is a modified form of Figure 3 in the original publication of Landersdorfer and Nation, 2021.

One approach involved the development of novel MIC-independent PK-PD metrics based upon quantification of the cumulative area under the antibiotic concentration-time curve and the cumulative area between the growth control and the bacterial-killing and -regrowth curves from *in vitro* time-kill experiments (Seeger et al., 2021a). While the potential applicability of this approach warrants further examination, it is not clear how other factors that may influence clinical outcome (e.g., immune function, severity of overall illness) can be incorporated. More attention has been directed at the use of mechanism-based (semi-mechanistic) modeling to describe and predict the full time-course of bacterial growth, killing, and resistance emergence in response to different antibiotic exposure profiles, an approach that is gaining in popularity (Bulitta et al., 2011; Nielsen et al., 2011; Landersdorfer et al., 2013; Mohamed et al., 2016; Rao et al., 2018; de Miranda Silva et al., 2019; Aranzana-Climent et al., 2020; Onufrak et al., 2020; Seeger et al., 2021b). Such models are usually based upon data from *in vitro* static and/or dynamic time-kill studies and can incorporate information on the time-course of effect on not only the total bacterial population but also resistant (less-susceptible) subpopulations. These models can enable translation to the clinic by being interfaced with a population PK model for the relevant patient group to predict the influence of established covariates of PK exposure (e.g., renal function, body size) on bacterial killing and development of resistance for antibiotic monotherapies and combination treatments, and also suggest dosing regimens for clinical evaluation (Bergen et al., 2016; Yadav et al., 2017; Yadav et al., 2019; Friberg, 2021). Beyond translation, mechanism-based models also have enormous potential for application in optimising the care of individual patients *via* model-informed precision dosing (MIPD) (Friberg, 2021; Landersdorfer and Nation, 2021; Wicha et al., 2021). Such an approach would be enhanced by having timely access to MIC-independent information on the characteristics of the bacterial strain causing infection, to gauge its antibiotic susceptibility. Fortunately, recognition of the clinical potential of MIPD coincides with a very large increase in the number of rapid molecular, genotypic and phenotypic (non-MIC) methods that are either already available or in development to assess antibiotic resistance mechanisms and susceptibility (Giacobbe et al., 2020; van Belkum et al., 2020). Mechanism-based models used in MIPD can also integrate information on the time-course of pathophysiological-induced changes in PK, in addition to the time-courses of the host immune response, biomarkers of infection status and clinical metrics indicating the severity of illness (Diep et al., 2018; Thorsted et al., 2020). Such an integrated package, when combined with real-time monitoring of antibiotic PK exposure and an adaptive feedback control system, has the potential to power a MIPD system (Figure 4). This would enhance the achievement of an optimal exposure and PK profile shape in an individual patient, for either monotherapy or combination regimens (Friberg, 2021; Landersdorfer and Nation, 2021; Wicha et al., 2021).

The three traditional MIC-based PK-PD indices have brought us a long way over the last several decades and will continue to find application for some time. However, it is important to move beyond those empirical metrics and more fully explore scientifically robust approaches of linking the time-course of exposure to an antibiotic with the time-course of its antibacterial effect, while also having the ability to integrate other factors that can influence treatment outcome in an infected patient. Looking to the future, a wonderful opportunity and challenge for the field of antibiotic PK-PD will be to continue to develop, refine and evaluate the translational and clinical application of mechanism-based models and other approaches aimed at streamlining the development of new antibiotics and optimizing the dosing of new and old antibiotics in individual patients. Success in this endeavor would be expected to make a wonderful contribution to efforts aimed at improving survival of individual patients, while addressing the ever-present threat of resistance and preserving the activity and clinical utility of precious antibiotics into the future.
